# Fibril treatment changes protein interactions of tau and α-synuclein in human neurons

**DOI:** 10.1016/j.jbc.2023.102888

**Published:** 2023-01-10

**Authors:** Tagan A. Griffin, Paul D. Schnier, Elisa M. Cleveland, Robert W. Newberry, Julia Becker, George A. Carlson

**Affiliations:** 1 Institute for Neurodegenerative Diseases, Weill Institute for Neurosciences, University of California, San Francisco, California, USA; 2 Department of Neurology, Weill Institute for Neurosciences, University of California, San Francisco, California, USA; 3 Department of Pharmaceutical Chemistry, University of California, San Francisco, California, USA

**Keywords:** α-synuclein, tau, prion, mass spectrometry, proteomics, BioID, proximity biotinylation, interactome, NGN2 neurons, 14-3-3, AD, Alzheimer’s disease, AP-MS, affinity purification MS, BioID2, biotin identification 2, CMVe, cytomegalovirus enhancer, FDR, false discovery rate, FTD, frontotemporal dementia, GO, Gene Ontology, GSK-3β, glycogen synthase kinase-3 beta, hESC, human embryonic stem cell, HRP, horseradish peroxidase, MAP2, microtubule-associated protein 2, MS, mass spectrometry, NDUFS8, NADH:ubiquinone oxidoreductase core subunit S8, *NGN2*, neurogenin-2, PD, Parkinson’s disease, PFF, preformed fibril, PrP, prion protein, RT-QuIC, real-time quaking-induced conversion, STRING, Search Tool for the Retrieval of Interacting Genes/Proteins, TEAB, triethylammonium bicarbonate, ThT, thioflavin T, TMT, tandem mass tag

## Abstract

In several neurodegenerative disorders, the neuronal proteins tau and α-synuclein adopt aggregation-prone conformations capable of replicating within and between cells. To better understand how these conformational changes drive neuropathology, we compared the interactomes of tau and α-synuclein in the presence or the absence of recombinant fibril seeds. Human embryonic stem cells with an inducible neurogenin-2 transgene were differentiated into glutamatergic neurons expressing (1) WT 0N4R tau, (2) mutant (P301L) 0N4R tau, (3) WT α-synuclein, or (4) mutant (A53T) α-synuclein, each genetically fused to a promiscuous biotin ligase (BioID2). Neurons expressing unfused BioID2 served as controls. After treatment with fibrils or PBS, interacting proteins were labeled with biotin *in situ* and quantified using mass spectrometry *via* tandem mass tag labeling. By comparing interactions in mutant *versus* WT neurons and in fibril- *versus* PBS-treated neurons, we observed changes in protein interactions that are likely relevant to disease progression. We identified 45 shared interactors, suggesting that tau and α-synuclein function within some of the same pathways. Potential loci of shared interactions include microtubules, Wnt signaling complexes, and RNA granules. Following fibril treatment, physiological interactions decreased, whereas other interactions, including those between tau and 14-3-3 η, increased. We confirmed that 14-3-3 proteins, which are known to colocalize with protein aggregates during neurodegeneration, can promote or inhibit tau aggregation *in vitro* depending on the specific combination of 14-3-3 isoform and tau sequence.

Tauopathies and synucleinopathies are characterized by the accumulation of misfolded tau or α-synuclein in the brain. Misfolded conformations of tau and α-synuclein can replicate autocatalytically *via* a mechanism shared with prion protein (PrP), whereby normally folded proteins are recruited into misfolded structures by templating (1). The misfolding process generates novel conformations with novel functions, driving toxicity and cell-to-cell transmission through oligomer growth and fragmentation (2, 3, 4). Particular misfolded conformations, or strains, are implicated in specific neurodegenerative diseases. For example, tau strains differ in Alzheimer’s disease (AD) and frontotemporal dementias (FTDs), and different strains of α-synuclein can cause Parkinson’s disease (PD) and multiple system atrophy (5, 6, 7, 8, 9, 10). Each strain can potentially engage in unique pathological interactions within the cell. Protein interactors such as glycogen synthase kinase-3 beta (GSK-3β) can influence both tau and α-synuclein pathology (11), as can direct interactions between tau and α-synuclein (12). Identifying additional shared interactors may lead to a better understanding of the mechanisms of neurodegeneration and point toward therapeutic targets for multiple diseases, including synucleinopathies and tauopathies.

Tau is a neuronal protein whose primary function is to regulate the stability of microtubules. Each of the six tau isoforms found in the brain has either three or four microtubule-binding repeat domains, designated R, in the C-terminal region and zero, one, or two amino-terminal domains, designated N, in the N-terminal region of tau (13). Tau isoforms are named based on the numbers of R and N domains in the protein; for example, 0N4R tau does not have N-terminal domains but has four C-terminal repeat domains, 2N3R tau has two N-terminal domains and three microtubule-binding repeat domains, and so on. While distinct functions of the individual domains are not completely understood, tau isoforms are known to be differentially expressed depending on age, developmental stage, and brain region. Microtubule dynamics and intracellular localization are also influenced by specific tau isoforms. Some tauopathies are distinguished by isoforms that accumulate in disease, such as 3R tau in Pick’s disease and 4R tau in progressive supranuclear palsy (14). For our study, we selected WT 0N4R tau and 0N4R tau with the destabilizing P301L mutation and directly compared their interactomes. P301L mutant tau is linked to FTD and is much more prone to aggregation and replication than WT tau.

In tauopathies such as AD and FTDP-17, tau is hyperphosphorylated and disengaged from microtubules. Tau misfolds into fibrils with conformations and isoform compositions that are unique to each disease (9, 15). Repeat domains in tau are sufficient to induce tau aggregation and fibril formation. In our study, a recombinant tau fragment containing only the four microtubule-binding and P301L-mutant repeat domains as a core (designated K18) was used to induce cytoplasmic tau aggregation and neurofibrillary tangles in human embryonic stem cell (hESC)–derived neurons.

α-Synuclein is another protein capable of adopting self-templated prion conformations. While many functions of α-synuclein remain unresolved, this membrane-associated intracellular protein is localized primarily in neuronal presynaptic compartments where it serves to regulate the clustering and release of synaptic vesicles (16). Similar to tau, α-synuclein can misfold, redistribute to cell bodies, and form dense aggregates known as Lewy bodies that are characteristic of PD and dementia with Lewy bodies. α-Synuclein is more prone to aggregation and amyloid formation than tau. We used WT α-synuclein preformed fibrils (PFFs) to induce aggregation in hESC-derived neurons in the current study. PFFs were formed by incubating purified α-synuclein monomers at 37 °C with shaking to produce aggregates (17). Unlike WT tau, WT α-synuclein can aggregate into PFFs, but the A53T familial PD-linked A53T mutation also used in these studies accelerates aggregation of α-synuclein.

There is accumulating evidence that these two proteins can influence the misfolding of one another. For example, more than half of postmortem AD cases were found to have α-synuclein pathology (18), whereas about half of PD cases have tau pathology (19). Evidence from genome-wide association studies shows that members of the tau (microtubule-associated protein tau) haplogroup H1 have an increased risk of both tauopathies and synucleinopathies (20, 21). Whether the relationship between tau and α-synuclein pathology is mediated by direct interactions or by shared interactors in a common environment is unclear.

There have been several MS-based interactome experiments that identified coaggregating or coimmunoprecipitating partners of tau and α-synuclein under physiological and pathological conditions (22, 23, 24, 25, 26). Cell lines, transgenic mouse neurons, and human cadavers have all been used as source material. Recently, Tracy *et al.* used two complementary methods, affinity purification MS (AP-MS) and proximity biotinylation MS, to identify WT and mutant tau interactors in neurons derived from human-induced pluripotent stem cells. In the proximity biotinylation approach, 2N4R tau was fused to an engineered ascorbate peroxidase, tagging proximal interactors with biotin that were subsequently identified by MS (ascorbate peroxidase MS); this revealed many cytoskeletal and synaptic interactors (26). To elucidate mechanisms common to tau and α-synuclein aggregation, we used a similar proximity biotinylation approach to compare the interactomes of WT and mutant (P301L) 0N4R tau to the interactomes of WT and mutant (A53T) α-synuclein in hESC-derived neurogenin-2 (*NGN2*) neurons. We added oligomerized recombinant fibrils to induce aggregation and model a prion-like replication process: 4R repeat domain tau (K18) or WT α-synuclein PFFs.

Using streptavidin beads, we purified interacting proteins from transgenic *NGN2*-hESC lines expressing a biotin ligase fused to the C terminus of WT tau, P301L tau, WT α-synuclein, A53T α-synuclein, or the biotin ligase alone. We then used MS to compare the relative abundance of streptavidin-purified and biotin-tagged proteins in each line at 24 h and 3 weeks after the addition of fibrils or PBS. For our experiments, we chose BioID2 (biotin identification 2), a mutated (R40G) promiscuous biotin ligase derived from *Aquifex aeolicus* (27). BioID2 generates biotinyl-5′-AMP in the presence of excess biotin, covalently labeling any lysine within ∼10 nm of the enzyme and permanently biotinylating interactors of the fused protein of interest (28). This powerful approach is particularly well suited to analyzing detergent-insoluble interactors because biotinylated proteins can be completely denatured before streptavidin-based purification.

We used a single *NGN2*-hESC clone containing an inducible promoter (TRE3G) driving an *NGN2* transgene, which in the presence of doxycycline rapidly converts ESCs into forebrain glutamatergic neurons (29). We fused tau and α-synuclein constructs to BioID2 and used those constructs to make four different cell lines expressing tau[WT]-BioID2, tau[P301L]-BioID2, α-synuclein[WT]-BioID2, or α-synuclein[A53T]-BioID2. As a control for nonspecific biotinylation, we made an additional *NGN2*-hESC line expressing BioID2 alone. Neurons expressing tau-BioID2 were treated with K18 or PBS, and neurons expressing α-synuclein-BioID2 were treated with PFFs or PBS; the cells were then harvested 24 h or 3 weeks postseeding. We found a set of proteins that interact with both tau and α-synuclein, predominantly under non–fibril-treated conditions (43 of 45 shared interactors) and followed up on a potential connection between 14-3-3 proteins and the replication of particular tau conformations.

## Results

### Generation of hESC-derived neurons expressing 0N4R tau-BioID2 or α-synuclein-BioID2

**Figure 1:**
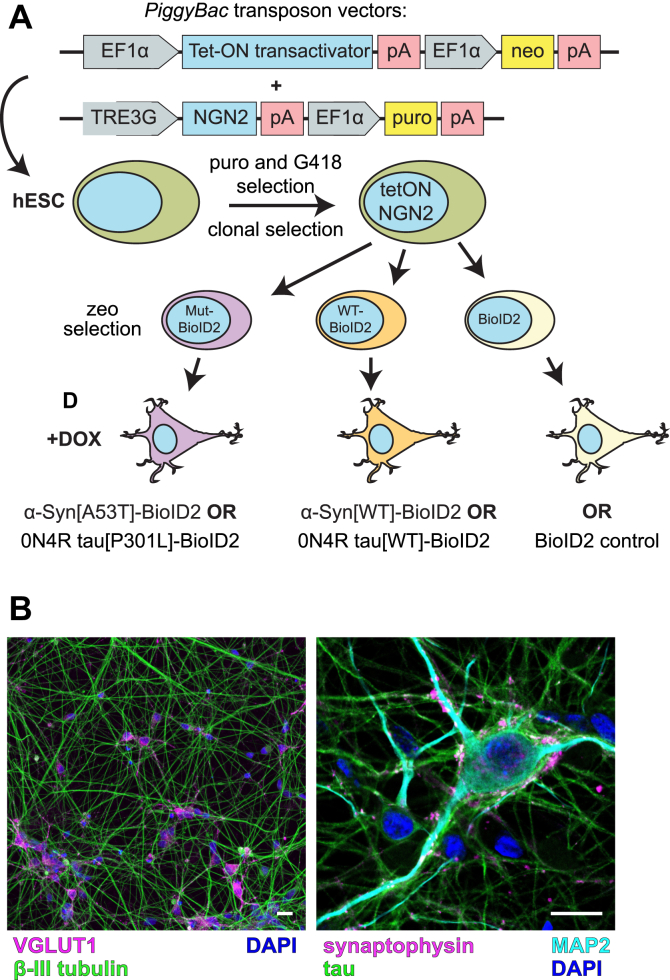
**Production and characterization of hESC-derived human neurons expressing BioID2-tagged tau or BioID2-tagged α-synuclein or controls expressing BioID2 alone.***A*, schematic illustrating the stepwise integration of *piggyBAC* transposon constructs into hESCs to generate human neurons expressing 0N4R tau[WT or P301L]-BioID2, α-synuclein[WT or A53T]-BioID2, or BioID2-only controls (CONT). *B*, immunofluorescent images of *NGN2*-neurons (no BioID2 transgene) 6 weeks postdifferentiation. The antibodies shown in each image include markers of glutamatergic neurons. The scale bar represents 20 μm. α-Syn, α-synuclein; BioID2, biotin ligase; DAPI, 4′,6-diamidino-2-phenylindole; DOX, doxycycline; EF1α, eukaryotic translation elongation factor 1α; hESC, human embryonic stem cell; MAP2, microtubule associated protein 2; MUT, mutant; *NGN2*, neurogenin-2; pA, poly(A) tail; puro, puromycin; TRE3G, Tet-ON 3G transactivator; VGLUT1, vesicular glutamate transporter 1; zeo, zeocin.

To identify tau and α-synuclein interactors in a disease-relevant cell type, we adapted recently developed techniques enabling the large-scale generation of cortical neurons from human pluripotent stem cells (hESCs (30, 31, 32)). Using *piggyBac* transposon vectors, we simultaneously introduced an inducible *NGN2* transgene and a constitutively expressed Tet-ON 3G transactivator into hESCs (Fig. 1*A*). Following drug selection and cloning, we obtained cells that grew well under standard hESC conditions but differentiated rapidly and uniformly into synaptophysin^+^/vesicular glutamate transporter 1^+^ excitatory neurons upon replating in neuronal media with doxycycline (Fig. 1*B*).

**Figure 2:**
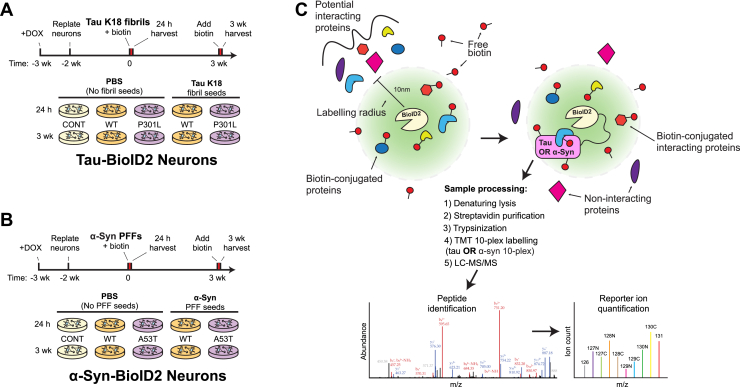
**Experimental timelines and illustration of the ten conditions compared in this study.** Experimental timelines of the experimental conditions compared in this study for *A*, 0N4R tau[WT]-BioID2 and 0N4R tau[P301L]-BioID2 neurons with and without K18 fibril seeds, and for *B*, α-Syn[WT]-BioID2 and α-Syn[A53T]-BioID2 neurons with and without α-synuclein PFFs. Tau and α-synuclein neurons are shown separately to emphasize that each of the ten conditions represented by a tissue culture dish was a separate trial to biotinylate and identify interacting proteins. *C*, schematic representing the biotinylation of interacting proteins by BioID2-conjugated tau or α-synuclein. Cellular proteins directly binding to BioID2-conjugated tau or α-synuclein (*pink shapes*) or coming within 10 nm of a BioID2-conjugated protein are biotinylated and classified as interacting proteins (*blue*, *orange*, and *yellow shapes*), unless similar biotinylation occurs in control neurons expressing free and unconjugated BioID2, indicating nonspecific binding. *Red circles* symbolize biotin that can be linked by BioID2 to cellular proteins interacting with or in close proximity (<10 nm) to a BioID2 fusion protein. Noninteractors (*magenta* and *purple shapes*) that are not within 10 nm of BioID2 are also depicted. Schematic also includes the workflow enabling quantitative comparisons of biotinylated proteins between experimental conditions. α-Syn, α-synuclein; BioID2, biotin ligase; CONT, control; DOX, doxycycline; MUT, mutant; PFF, preformed α-synuclein fibril; TMT, tandem mass tag; wk, week.

Using a single *NGN2*-hESC clone, we introduced an additional *piggyBac* vector expressing one of the following: 0N4R tau[WT]-BioID2, 0N4R tau[P301L]-BioID2, α-synuclein[WT]-BioID2, α-synuclein[A53T]-BioID2, or BioID2 alone. All these were under the control of a cytomegalovirus enhancer (CMVe)/synapsin promoter to drive expression preferentially in differentiated neurons. We chose BioID2, a mutated biotin ligase derived from *A. aeolicus*, for its relatively small size (∼25 kDa) and capacity for efficient biotinylation (27). Polyclonal BioID2 *NGN2*-hESC lines were selected with zeocin and expanded prior to differentiation. To initiate neuronal differentiation, we dissociated and replated the immature cells in doxycycline-containing differentiation media. After 5 days, we dissociated the immature neurons again and replated them in terminal differentiation media 2 weeks prior to the addition of fibrils and/or biotin (Fig. 2, *A* and *B*). Immature neurons could also be frozen following dissociation rather than directly replated, which may be useful for having a supply of neurons for high-throughput screening applications.

### Spontaneous and seeded tau and α-synuclein aggregation in neurons

**Figure 3:**
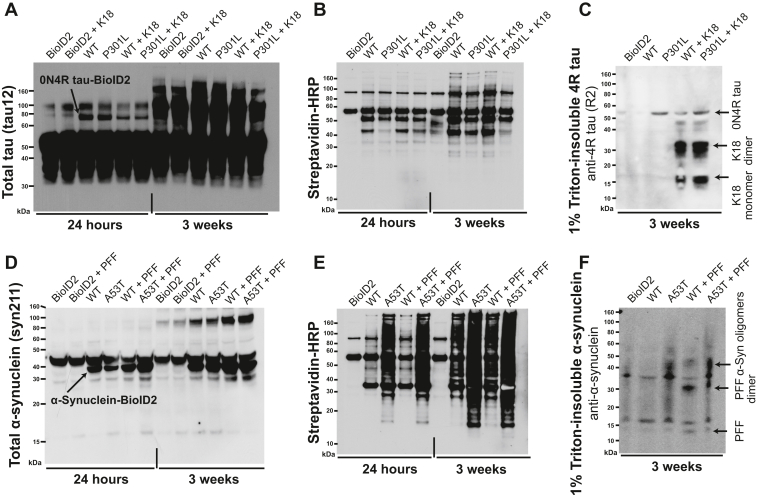
**Expression of tau-BioID2 and α-synuclein in hESC-derived neurons.** Western blots for *A*, total tau and for *D*, total α-synuclein using syn211 antibody in neuron lysates 24 h and 3 weeks after fibril or PBS treatment. The *arrows* mark the expected sizes of the BioID2 fusion proteins. *B* and *E*, streptavidin–HRP blots of neuron lysates labeling all biotinylated proteins 24 h or 3 weeks postfibril treatment. *C*, Western blot for 1% Triton-insoluble 4R tau using an in-house polyclonal antibody against the R2 region. *F*, Western blot for total α-synuclein in the 1% Triton-insoluble fractions of neuron lysates 3 weeks after fibril treatment. α-Syn, α-synuclein; BioID2, biotin ligase; hESC, human embryonic stem cell; HRP, horseradish peroxidase; K18, recombinant 4R repeat domain P301L fibril; PFF, preformed α-synuclein fibril.

Two weeks after replating, we added recombinant 4R tau repeat-domain fibrils (K18) or PBS to the neurons expressing tau-BioID2 and recombinant PFFs or PBS to the neurons expressing α-synuclein-BioID2 (17, 33). We added exogenous biotin either simultaneously with or 3 weeks later than the fibrils to initiate labeling of tau and α-synuclein interactors (Fig. 2, *A* and *B*). Including control cells expressing free BioID2 (not conjugated to either α-synuclein or tau), there were ten conditions each for α-synuclein and tau to identify interacting proteins by proximity biotinylation; an overview of the affinity biotinylation process is illustrated in Figure 2*C*. We then harvested the neurons in 8 M urea 24 h after the addition of biotin. We confirmed expression of the tau and α-synuclein BioID2 transgenes *via* antitotal tau or antitotal α-synuclein Western blots of the lysates (Fig. 3, *A* and *D*). We confirmed the functionality of the BioID2 enzymes *via* a streptavidin–horseradish peroxidase (HRP) blot, which labels all biotinylated proteins (Fig. 3, *B* and *E*).

**Figure 4:**
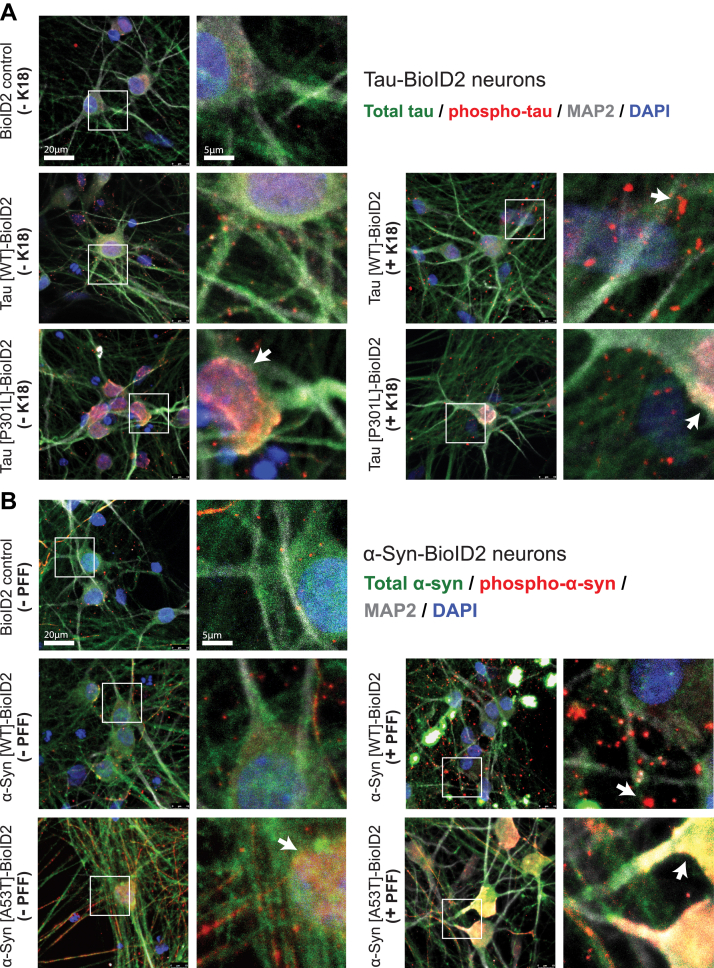
**Immunofluorescent staining of tau and α-synuclein in fibril-treated neurons.** Immunofluorescent images of total tau or α-synuclein (tau12 and syn211) in neurons expressing tau-BioID2 or α-synuclein-BioID2. All images were taken 3 weeks after treatment with fibrils or PBS. *A*, disease-associated phospho-tau (pS202/pT205/pS208, AT8) is shown in *red*. *Arrows* indicate AT8^+^ puncta in WT neurons and strong AT8 staining in P301L cell bodies. *B*, similar images of WT and A53T α-syn-BioID2. Disease-associated phospho-α-syn (pS129, EP1536Y) is shown in *red*. *Arrows* indicate large phospho-α-syn^+^ puncta in WT neurons and strong phospho-α-syn staining in A53T cell bodies. α-Syn, α-synuclein; BioID2, biotin ligase; DAPI, 4′,6-diamidino-2-phenylindole; K18, recombinant 4R repeat domain P301L fibrils; MAP2, microtubule-associated protein 2; PFF, preformed α-synuclein fibril.

To test for the presence of insoluble tau or α-synuclein, we performed detergent extractions and Western blots. We ran a parallel set of seeding experiments in which we lysed the neurons in PBS, rather than denaturing them in 8 M urea, to detect insoluble tau and α-synuclein 3 weeks after the addition of K18 or PFF seeds. After 1% Triton X-100 detergent extraction followed by ultracentrifugation, we identified detergent-insoluble 4R tau or total α-synuclein by Western blot (Fig. 3, *C* and *F*). Two prominent bands corresponding to monomeric and dimeric forms of the recombinant K18 seeds were observed in K18-treated neurons. An additional band corresponding to 0N4R tau was present in the seeded neurons expressing tau[WT]-BioID2, whereas there was no 0N4R band in unseeded neurons expressing tau[WT]-BioID2. A band corresponding to 0N4R tau was seen in both seeded and unseeded neurons expressing tau[P301L]-BioID2 (Fig. 3*C*). Oligomeric α-synuclein bands were observed in both seeded and unseeded neurons expressing α-synuclein[A53T]-BioID2. Similar bands were also present in seeded, but not unseeded, neurons expressing α-synuclein[WT]-BioID2. The tau and α-synuclein band patterns were different from those in control neurons expressing only BioID2 (Fig. 3*F*). The results suggest that tau[WT]-BioID2 and α-synuclein[WT]-BioID2 aggregate in response to K18 fibrils or PFFs. They also suggest that mutant tau[P301L]-BioID2 and mutant α-synuclein[A53T]-BioID2 aggregate even in the absence of fibrils, which is likely because of a greater propensity of the mutant forms of the proteins to aggregate than the WT forms. We did not see obvious bands corresponding to the tau-BioID2 fusion proteins, suggesting that endogenous 0N4R WT tau (or a truncated BioID2 fusion) was the predominant aggregated species. In the α-synuclein-BioID2 detergent-insoluble blots, it is unclear whether the extra bands derive from the BioID2 fusion proteins or from oligomeric forms of endogenous α-synuclein. Confocal images of the neurons stained with antibodies against total and phospho-tau (Fig. 4*A*) or total and phospho-α-synuclein (Fig. 4*B*) were consistent with the Western blot results.

### Identification and quantification of biotinylated interactors

**Figure 5:**
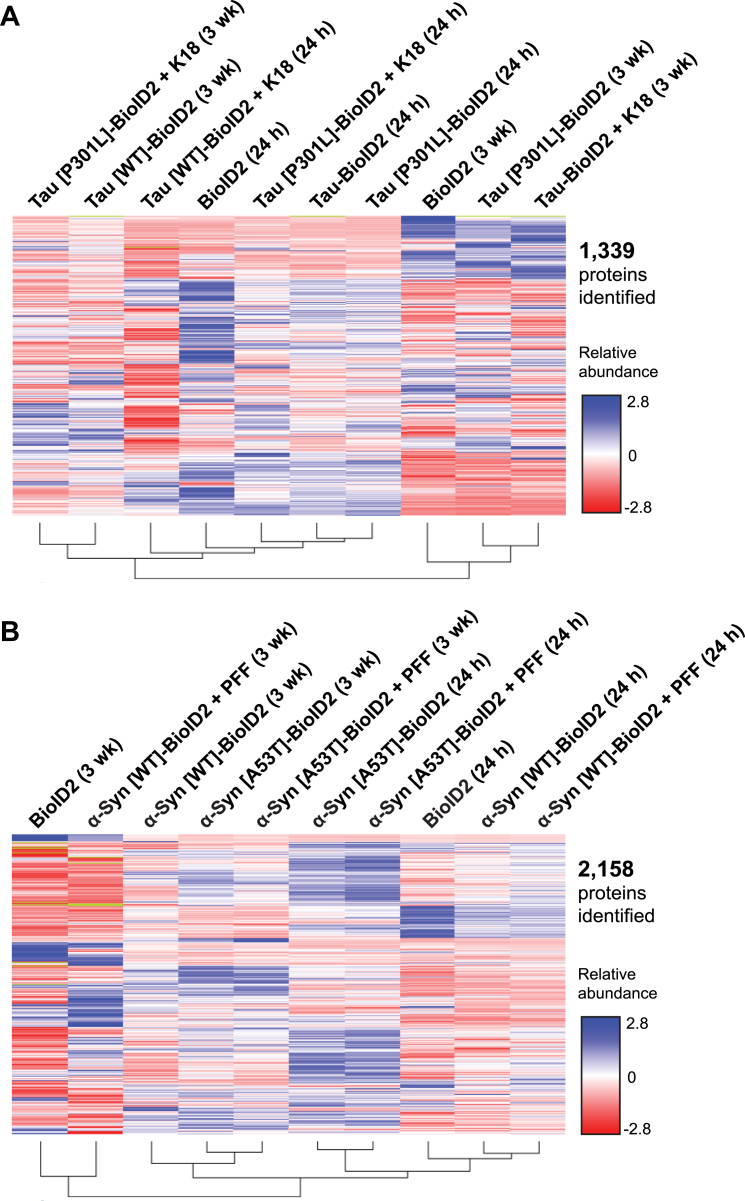
**Heatmaps of biotinylated proteins identified in tau-BioID2 and α-synuclein-BioID2 neurons.***A*, Tau-BioID2 and *B*, α-synuclein-BioID2 TMT10plex data represented by heatmaps and clustered according to similarity. Colors represent the relative abundance of TMT reporter ions corresponding to each identified protein (average of three technical replicates). There were 1339 quantifiable proteins identified in the tau 10plex sample and 2158 quantifiable proteins identified in the α-synuclein 10plex sample. α-Syn, α-synuclein; BioID2, biotin ligase; K18, recombinant 4R repeat domain P301L fibrils; PFF, preformed α-synuclein fibril; TMT, tandem mass tag; wk, week.

**Figure 6:**
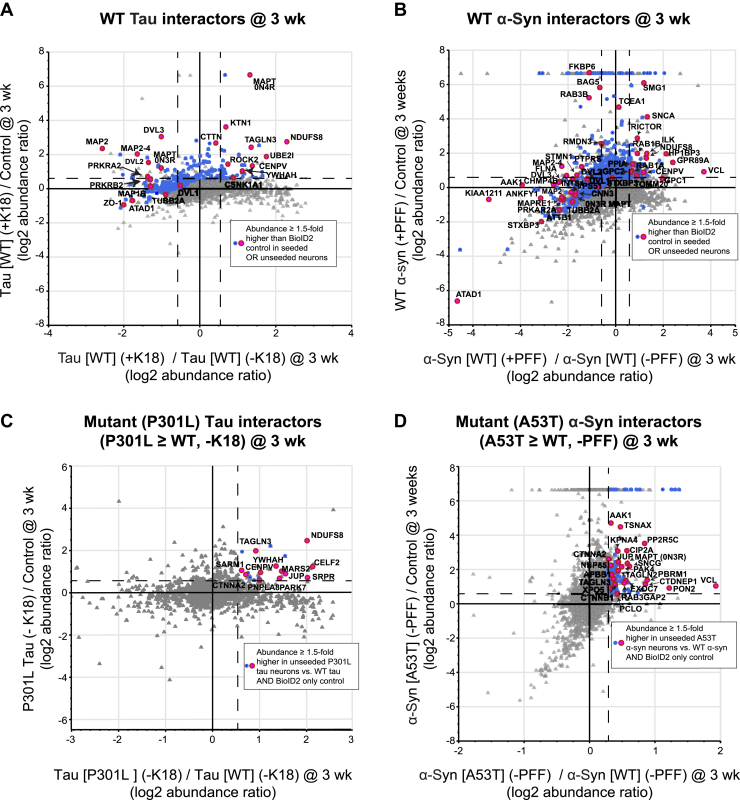
**Abundance of biotinylated proteins in fibril-treated *versus* fibril-untreated WT neurons and in mutant *versus* WT neurons.** Plots displaying the abundance (3 weeks postfibrils) of biotinylated proteins in WT neurons. Abundance in *A*, tau[WT]-BioID2 neurons or *B*, α-synuclein[WT]-BioID2 neurons relative to the BioID2-only control was plotted on the *y*-axis, and the abundance in fibril-treated relative to untreated neurons was plotted on the *x*-axis. Labeled *blue* and *pink dots* represent interactors that met the ≥1.5-fold abundance threshold (over BioID2-only control) in either fibril-treated or fibril-untreated neurons at 3 weeks postseeding. Plots displaying the abundance of mutant-specific interactors. The abundance of biotinylated proteins in untreated *C*, mutant tau-BioID2 or *D*, α-synuclein-BioID2 neurons was plotted relative to their abundance in the BioID2-only control (*y*-axis) and relative to their abundance in WT neurons (*x*-axis). Labeled *blue* and *pink dots* represent proteins ≥1.5-fold more abundant in mutant neurons relative to WT and BioID2-only control neurons. α-Syn, α-synuclein; BioID2, biotin ligase; K18, recombinant 4R repeat domain P301L fibrils; PFF, preformed α-synuclein fibril; wk, week.

Following lysis in 8 M urea, we purified biotinylated proteins from denatured neuron lysates with magnetic streptavidin beads. After extensive washing, the purified proteins were digested by trypsin on-bead, and the peptides were labeled with amine-reactive tandem mass tag labels (TMT 10-plex) for quantitative MS. We then organized the samples into one group for tau and one group for α-synuclein with two BioID2-only control samples included in each group. We combined the samples from each group and subjected them to LC–MS analysis. Following three replicate injections, we combined and analyzed the data. The SEQUEST HT peptide search engine identified 1339 quantifiable proteins in the tau group (Fig. 5*A*) and 2158 quantifiable proteins in the α-synuclein group (Fig. 5*B*) with a false discovery rate (FDR) of <1%. Each quantified protein was associated with a set of TMT ion abundance values, reflecting the relative protein abundance in each of the ten samples in the tau group and in each of the ten samples in the α-synuclein group (Fig. 5). We then plotted the abundance values of identified proteins between samples within the tau group or between samples within the α-synuclein group (Fig. 6).

To identify changes in tau-interacting and α-synuclein–interacting proteins in response to fibril seeding, we plotted each protein's abundance in fibril-treated *versus* fibril-untreated neurons on the *x*-axis against the ratio of each protein’s abundance in seeded neurons *versus* BioID2-only control neurons on the *y*-axis (Fig. 6, *A* and *B*). We visualized how the abundance of biotinylated proteins responded to the addition of fibrils by marking the data points of all proteins that were ≥1.5-fold more abundant in fibril-treated or fibril-untreated neurons relative to BioID2-only control neurons. 0N4R tau and α-synuclein were among the most abundant proteins in fibril-treated neurons relative to both untreated neurons and BioID2-only control neurons 3 weeks postfibril treatment, which validated our overall approach.

We compared the abundance of each protein in the tau-BioID2 and α-synuclein-BioID2 samples (WT and mutant) to its abundance in the BioID2-only controls at 24 h and 3 weeks postseeding. We considered only proteins with ≥1.5-fold higher abundance relative to both BioID2-only controls to be interacting proteins. We assembled a list of ratios representing the relative abundance of interacting proteins in fibril-treated *versus* fibril-untreated neurons along with an approximation of that protein’s total abundance (number of peptide spectral matches) in the tau-BioID2 or α-synuclein-BioID2 groups (File S1).

To evaluate mutant-specific tau and α-synuclein interactomes, we plotted interactor abundance in unseeded tau[P301L]-BioID2 or α-synuclein[A53T]-BioID2 neurons relative to their unseeded WT counterparts (Fig. 6, *C* and *D*). Using a cutoff threshold of 1.5-fold, we identified interactors that were more abundant in unseeded mutant neurons relative to WT neurons. Surprisingly, the mutant and WT tau-BioID2 interactor abundance ratios (seeded *versus* unseeded) often pointed in opposite directions. For example, in fibril-treated tau[WT]-BioID2 neurons, microtubule-associated protein 2 (MAP2; the most abundant interactor overall) had the lowest abundance ratio (+K18/−K18 = 0.17) of all identified interactors, which is consistent with a loss of physiological binding between MAP2 and tau. But in neurons expressing tau[P301L]-BioID2, MAP2 had one of the highest abundance ratios (+K18/−K18 = 9.3) (File S1). Although MAP2 and tau are very similar proteins, they are not known to coaggregate and have been reported to prevent aggregation of one another (34). In the α-synuclein[A53T]-BioID2 neurons, very few interactors (∼3%) were differentially abundant in fibril- *versus* PBS-treated neurons. Because the interactor abundance ratio (fibril treated *versus* PBS treated) was likely biased by the spontaneous aggregation we observed, an additional list of mutant *versus* WT interactors was generated for tau and α-synuclein without regard to abundance ratio (File S1).

**Figure 7:**
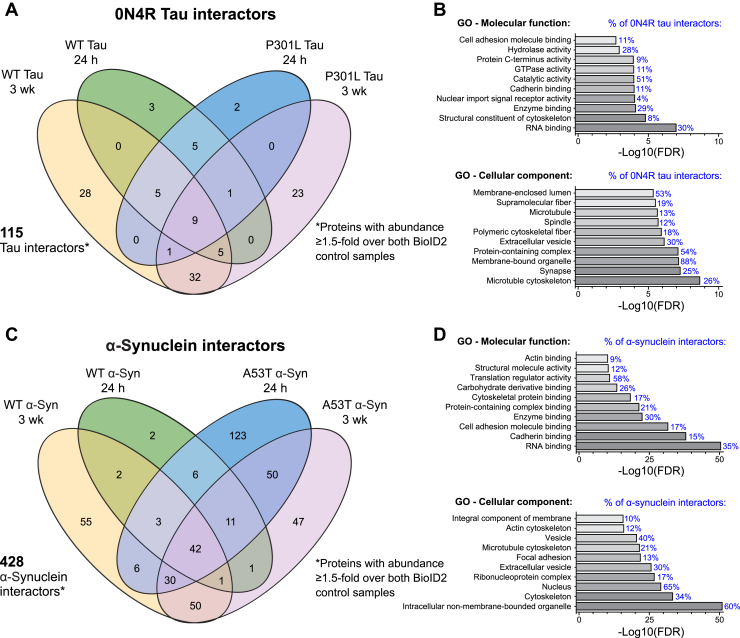
**Comparison of tau-BioID2 and α-synuclein-BioID2 interactors.** Venn diagrams showing the number of WT and mutant *A*, tau-BioID2 or *C*, α-synuclein-BioID2 interacting proteins identified at each time point. Proteins were included if they were present in either the fibril-treated or fibril-untreated neurons at ≥1.5-fold higher abundance than both BioID2-only controls (24 h and 3 weeks postseeding). Gene Ontology enrichment analysis of all *B*, tau-BioID2 and *D*, α-synuclein-BioID2 interactors. Enriched GO terms—molecular functions and cellular components—were determined by a PANTHER over-representation test. The top ten nonredundant terms were plotted with the accompanying −log10(FDR) plotted on the *x*-axis. The percentage of all tau-BioID2 or α-synuclein-BioID2 interactors associated with each GO term is listed adjacently in *blue*. α-Syn, α-synuclein; BioID2, biotin ligase; FDR, false discovery rate; GO, Gene Ontology; wk, week.

In total, we identified 115 proteins as tau-BioID2 interactors (Fig. 7*A*) including known tau interactors, such as tubulins, MAPs, and 14-3-3 proteins (Fig. 7*A* and File S1) (35, 36). Proteins identified as interactors of P301L but not WT tau included 14-3-3θ and DJ-1 (Parkinsonism-associated deglycase), a PD-related protein recently described as a P301L-specific tau interactor (File S1) (37). We identified 428 protein interactors among the α-synuclein-BioID2 samples, including Rab-3B, γ-synuclein, and the nucleoporins Nup85, Nup107, and Nup214 (Fig. 7*C* and File S1). A53T-specific α-synuclein interactors included GSK-3β and septins 3, 5, 7, 9, and 11 (File S1).

The most abundant biotinylated proteins with an abundance ratio ≥1.5 in seeded *versus* unseeded neurons in the tau[WT]-BioID2 and α-synuclein[WT]-BioID2 cells were tau and α-synuclein, respectively. We identified 0N3R tau as an interactor of both WT and A53T α-synuclein, but α-synuclein was not a hit in any of our mutant or WT 0N4R tau-BioID2 samples (File S1).

### Comparison of tau-BioID2 and α-synuclein-BioID2 interactomes

There was significant overlap between the tau-BioID2 and α-synuclein-BioID2 interactomes (Fig. 7). Using the PANTHER Gene Ontology (GO) statistical overrepresentation test (38), we found that the lists of tau-interacting and α-synuclein–interacting proteins were both significantly enriched for proteins related to cytoskeleton binding, extracellular vesicles, and RNA binding, among others (FDR ≤ 0.05) (Fig. 7, *B* and *D*). Of the 115 tau-BioID2 interactors and 428 α-synuclein-BioID2 interactors, 45 were the same. All shared interactors were reduced in fibril-treated neurons, except for two proteins (NADH:ubiquinone oxidoreductase core subunit S8 [NDUFS8] and centromere protein V), implying some loss of shared physiological interactors.

**Figure 8:**
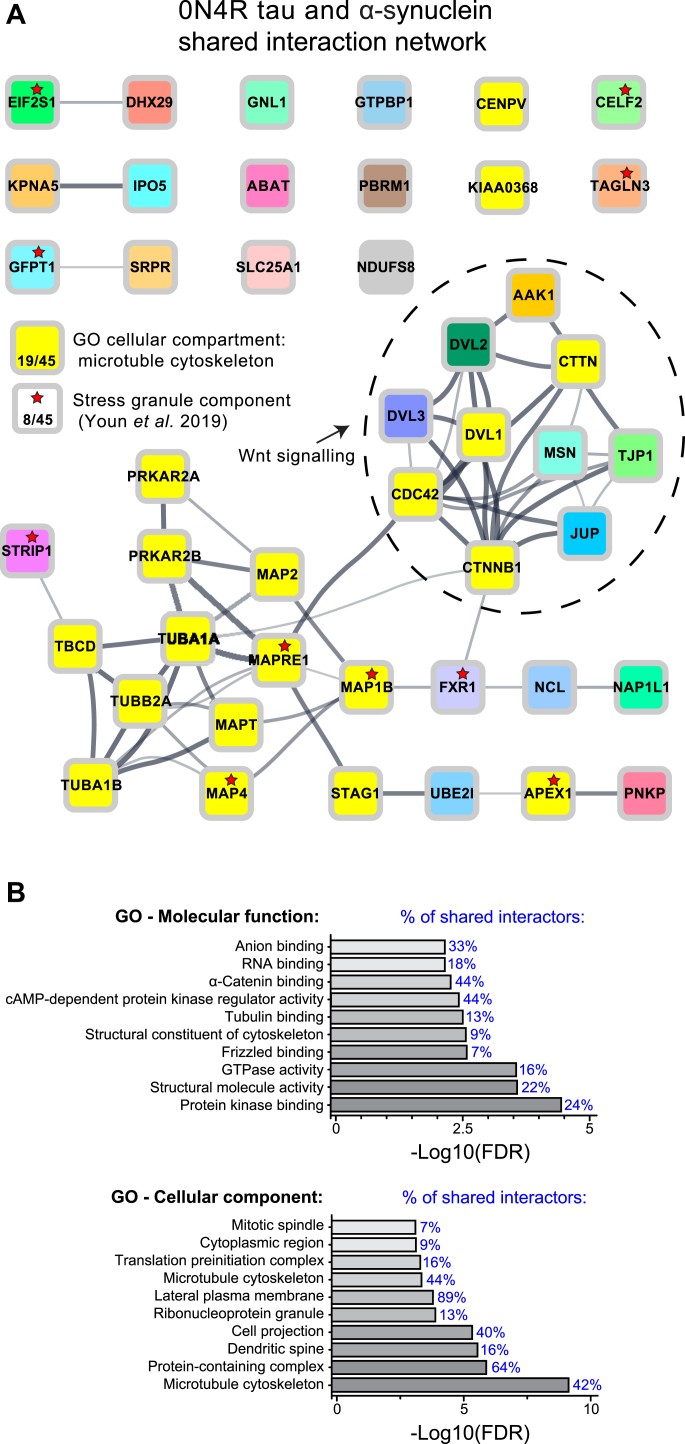
**Interactome network of shared tau-BioID2 and α-synuclein-BioID2 interactors.** Forty-five proteins were identified as being interactors of both tau-BioID2 and α-synuclein-BioID2. *A*, a STRING protein–protein interaction network of the shared interactors was visualized using the StringApp in Cytoscape. Two prominent clusters emerged: one related to microtubules (19 of 45 proteins) and the other related to Wnt signaling (10 of 45 proteins). There were also a significant number of proteins (8 of 45) that are known components of RNA stress granules. *B*, the top ten most enriched GO terms (molecular function and cellular component) were plotted according to their FDR, and the percentage of shared interactors associated with each GO term is labeled in *blue*. Of all the tau-BioID2 and α-synuclein-BioID2 shared interactors, 89% were associated with the lateral plasma membrane. BioID2, biotin ligase; FDR, false discovery rate; GO, Gene Ontology; STRING, Search Tool for the Retrieval of Interacting Genes/Proteins.

To evaluate what role these shared interactors might play, we subjected the list of 45 proteins to GO analysis and Search Tool for the Retrieval of Interacting Genes/Proteins (STRING) analysis (39) (Fig. 8, *A* and *B*). GO analysis revealed that 18 of 45 shared interactors were associated with the GO term “microtubule cytoskeleton” and that 40 of 45 shared interactors were associated with the GO term “lateral plasma membrane” (Fig. 8, *A* and *B*). STRING interaction analysis revealed two clusters of interacting proteins: one related to the microtubule cytoskeleton and the other related to Wnt signaling. The ten shared Wnt-related interactors included beta-catenin, cell division cycle 42, and all three disheveled proteins (DVL-1, DVL-2, and DVL-3). Of the 45 shared interactors, eight have been previously identified as components of RNA granules (40). RNA granule–associated proteins were recently shown to mislocalize to cytoplasmic tau aggregates in the brain (41). One particularly intriguing interactor whose abundance was increased in fibril-treated tau-BioID2 and α-synuclein-BioID2 neurons was NDUFS8, a core mitochondrial complex I subunit. The increased abundance of biotinylated NDUSF8 in fibril-treated neurons suggests a potential mechanism for the mitochondrial dysfunction observed in neurodegenerative disease (42).

### Interaction of 14-3-3 protein isoforms with tau

**Figure 9:**
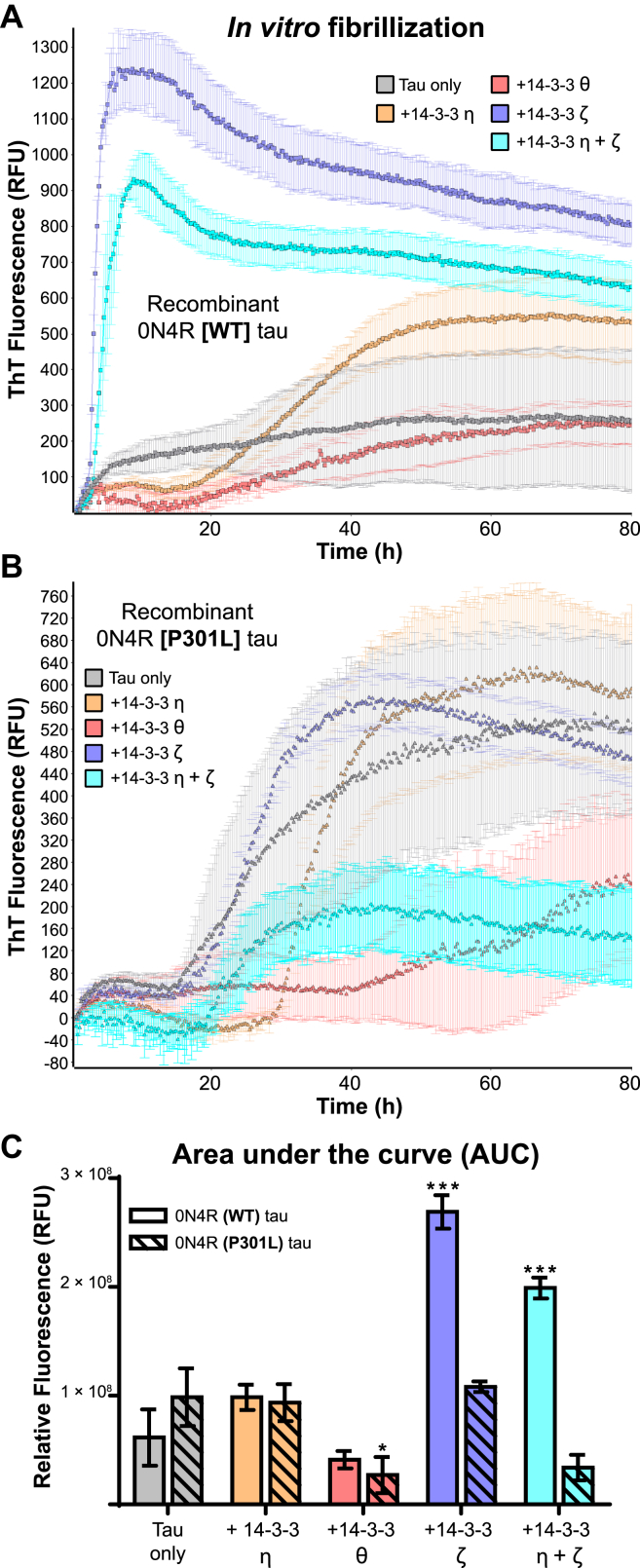
**14-3-3 isoforms differentially influence the fibrillization of recombinant WT and P301L 0N4R tau.** 0N4R tau amyloid formation monitored by RT-QuIC in the presence of 14-3-3 proteins. *A*, WT tau (10 μM) was incubated at 37 °C for 1 week in the presence of heparin (10:1 ratio of tau:heparin) with alternating cycles of shaking and rest. Recombinant 14-3-3 proteins (ƞ, θ, ζ, or ƞ + ζ) were added at 2 μM. Fibrillization was monitored with the amyloid-sensitive dye ThT. *B*, identical reaction conditions but with P301L 0N4R tau. *C*, quantification of ThT fluorescence (total area under the curve). Reactions containing 14-3-3 proteins were compared with the WT or P301L tau-only controls (∗*p* ≤ 0.05, ∗∗∗ *p* ≤ 0.01). Each sample was run in triplicate, and means and standard deviations of AUCs are displayed. AUC, area under the curve; RFU, relative fluorescence unit; RT-QuIC, real-time quaking-induced conversion assay; ThT, thioflavin T.

The second most abundant protein interactor with an abundance ratio ≥1.5 (fibrils *versus* no fibrils) in tau[WT]-BioID2 neurons was 14-3-3ƞ, which belongs to a promiscuous and highly conserved family of seven phosphoprotein adaptor proteins. In addition to 14-3-3ƞ, we found that tau[P301L]-BioID2 also interacted with 14-3-3θ. 14-3-3ζ was previously shown to stimulate aggregation of recombinant tau *in vitro* and transgenic tau in cell culture (43, 44). We hypothesized that interactions with 14-3-3 proteins may promote tau amyloid conversion in a tau sequence–specific and 14-3-3 isoform–specific manner. We tested this possibility in the real-time quaking-induced conversion (RT-QuIC) assay (45) using recombinant full-length WT or P301L 0N4R tau. Our reactions included the amyloid-sensitive dye thioflavin T (ThT) and heparin along with of one of three 14-3-3 isoforms either individually or in combination (ƞ, θ, ζ, or ƞ + ζ) (Fig. 9). We found that including 14-3-3 proteins in the fibrillization reactions had differential effects on ThT fluorescence depending on the 14-3-3 isoform and tau sequence. For example, relative to tau alone, 14-3-3ζ increased ThT fluorescence in the WT but not P301L tau reactions. The combination of 14-3-3ζ + ƞ also promoted ThT fluorescence in WT tau reactions, but the combination was not significantly different from 14-3-3ζ alone. 14-3-3ƞ did not significantly promote or inhibit ThT fluorescence in either the WT or P301L tau reactions. 14-3-3θ was unique among the 14-3-3 protein isoforms tested in its ability to significantly decrease ThT fluorescence in the P301L tau reactions (*p* < 0.05) (Fig. 9*C*). These results suggest that the 14-3-3 isoform composition may influence the susceptibility of some cell types to replication of particular tau strains.

## Discussion

Proximity-dependent BioID has emerged as one of the most useful techniques for delineating protein interactors, especially transient or insoluble interactors that would otherwise be missed by typical affinity-based approaches. By combining BioID with TMT labeling, we were able to quantify changes in the abundance of biotinylated tau and α-synuclein interactors following the addition of recombinant fibrils. The tau and α-synuclein interactors we identified were consistent with those identified in other interactome studies, including those done in cell lines, primary mouse neurons, induced pluripotent stem cell–derived neurons, and detergent-insoluble human tissue (22, 23, 26, 46). However, our study is unique in its direct comparison of fibril- *versus* PBS-treated neurons and in its comparison of tau and α-synuclein interactors in the same experimental system.

A recent report—utilizing both AP-MS and proximity biotinylation MS in *NGN2*-derived neurons—identified many of the same tau-interacting proteins found in our study, highlighting physiological tau functions at the cytoskeleton and synaptic membranes (26). One notable discrepancy between the two studies relates to mitochondrial interactors in neurons expressing WT *versus* mutant tau. We observed increased interactions between tau and the mitochondrial complex I protein NDUFS8 in unseeded neurons expressing P301L *versus* WT tau and in seeded *versus* unseeded neurons expressing WT tau. In contrast, Tracy *et al.* identified a number of mitochondrial complex I interactors (including NDUFS8) as less abundant in neurons expressing P301L *versus* WT tau using AP-MS. One potential explanation for this could be the different tau isoforms used (0N4R *versus* 2N4R), whereas another may lie in the method used to purify tau interactors. In their AP-MS experiments, Tracy *et al.* used anti-FLAG antibodies to pull down interactors along with FLAG-tagged tau. Unlike streptavidin-based methods that allow for protein denaturation prior to purification, antibody-based methods require proteins to be solubilized; however, aggregated tau interactors may not be accessible to antibodies in solution.

After treating neurons expressing WT tau or WT α-synuclein with recombinant fibrils, we observed that biotinylated 0N4R tau or α-synuclein was more abundant, whereas known interactors were less abundant. These results are consistent with a model of tau and α-synuclein prion replication involving the transcellular propagation and accumulation of misfolded proteins, with a concomitant loss of physiological interactions. Furthermore, we identified a direct interaction between 0N3R tau and α-synuclein in the neurons expressing WT and A53T α-synuclein. The observed decrease in biotinylated 0N3R tau in fibril-treated neurons expressing WT α-synuclein, combined with the absence of biotinylated α-synuclein in the neurons expressing 0N4R tau, suggests that isoform-specific tau–α-synuclein interactions may be involved in neurodegenerative disease progression. For example, both α-synuclein and the related β-synuclein can function as chaperones and stabilize each other and other proteins, like tau, to prevent their aggregation (47, 48). The findings that tau and α-synuclein interact and that pathological aggregates of tau and α-synuclein frequently appear together suggests that these interactions may indeed be important in neurodegenerative diseases. The earlier report by Lu *et al.* (12) that located the sites of interaction is compatible with the findings presented here.

Formation, transcellular propagation, and accumulation of misfolded proteins reflect dramatic conformational conversion of the native forms of tau and of α-synuclein into self-replicating prion conformations. This may be accompanied by concomitant loss of the ability to bind to proteins that interact with the native forms of tau and α-synuclein. The nature and origin of prion amyloid “seeds” that can initiate and perpetuate aggregation of tau and α-synuclein prions is uncertain. Some investigators view amyloid as inert or even protective (by sequestering toxic oligomers), whereas others consider tau or α-synuclein amyloid aggregates to be sources of seeds that enable the spread of misfolded proteins. It is also worth emphasizing that the identification of proteins using BioID2 is not restricted to proteins that are physically bound to the target protein; rather, BioID2 can label any protein within 10 nm, potentially including protein seeds or toxic oligomers released from fibrils.

By comparing tau and α-synuclein interactors using GO and STRING analysis, we identified microtubules and Wnt signaling complexes as potential loci of tau and α-synuclein interactions. α-Synuclein is not typically thought of as a microtubule-associated protein, but there is evidence that it can play an important role in the regulation of microtubules (49, 50, 51). For example, α-synuclein has been shown to stimulate activity of the tau kinase GSK-3β (11, 52), and tau phosphorylation by GSK-3β can induce tau amyloid formation (53). We identified all three disheveled proteins as interactors of both tau and α-synuclein. Disheveled cytoplasmic phosphoproteins regulate microtubule formation and the Wnt signaling pathway, which governs many cellular processes, including cell proliferation, survival, migration, differentiation, polarity, and stem cell renewal (54). Disheveled proteins mediate Wnt signaling through oligomerization, suggesting a mechanism by which tau and α-synuclein may play a role in Wnt-mediated synaptic dysfunction (55, 56).

In addition, we identified 14-3-3ƞ as a WT tau interactor and 14-3-3ƞ and θ as P301L tau interactors. Interestingly, 14-3-3 proteins colocalize with several neurodegenerative disease–associated proteins in a disease-specific and 14-3-3 isoform–specific manner. For example, plaques in the PrP prion disorder Creutzfeldt–Jacob disease colocalize with 14-3-3ζ, whereas plaques in the PrP prion disorder Gerstmann–Sträussler–Scheinker disease colocalize with 14-3-3ε (57, 58). Aggregates of superoxide dismutase 1 colocalize specifically with 14-3-3β and γ in patients with familial amyotrophic lateral sclerosis and in mice expressing mutant (G93A), but not WT, superoxide dismutase 1 (59). α-Synuclein in multiple system atrophy colocalizes with at least six 14-3-3 isoforms (60, 61, 62), whereas α-synuclein in PD does not colocalize with 14-3-3η, σ, or β (63). When α-synuclein was coexpressed with 14-3-3θ in primary mouse neurons, the toxicity of α-synuclein was decreased (64). The chaperone activity of 14-3-3 can prevent spontaneous amorphous aggregation or aggregation into amyloid fibrils (65, 66, 67). The different 14-3-3 isoforms can have varying chaperone activity against the same α-synuclein client protein (66), but only 14-3-3η and θ prevented α-synuclein aggregation, as monitored by atomic force microscopy. These earlier findings and the results presented here provided the impetus for testing 14-3-3 proteins individually and in combination. Finally, tau aggregates in AD preferentially interact with 14-3-3ζ (68), whereas tau aggregates in Pick’s disease contain all seven 14-3-3 isoforms in roughly equal proportions (69). Our experiments provide further evidence in support of specific 14-3-3–tau interactions. Given that different cell types in the brain express different subsets of 14-3-3 proteins, it is plausible that these proteins select, amplify, or inhibit specific strains, and this may help to explain the regional distribution seen in various neurodegenerative diseases.

## Experimental procedures

### Plasmid constructs

A *piggyBac* transposon expression plasmid (PB510B-1) was obtained from System Biosciences. The promoter was replaced with an inducible Tet-ON 3G promoter, a constitutive eukaryotic translation elongation factor 1α (EF1α) promoter, or a CMVe/synapsin promoter. In some plasmids, the puromycin resistance gene was replaced with a neomycin or zeocin resistance gene. The human *NGN2* transgene and BioID2 transgenes were codon optimized and synthesized (IDT) and then inserted into the expression plasmids *via* In-Fusion cloning (Takara). BioID2 was genetically fused to the C terminus of 0N4R (WT or P301L) tau or α-synuclein (WT or A53T) using a 15-amino acid linker that is compatible with tau and α-synuclein aggregation in cell lines expressing tau or α-synuclein fused to YFP (70).

### Generation and culture of *NGN2*-ESCs

hESCs (H1) were obtained from the WiCell Institute, cultured in StemPro media (Gibco) on Matrigel, and routinely passaged using Versene. To generate the inducible *NGN2* line, we electroporated ESCs (Nucleofector program A-023; Lonza) with three plasmids: *piggyBac*-EF1α::Tet-ON 3G transactivator, *piggyBac*-TRE3G::*NGN2*, and a transient Super *PiggyBac* transposase plasmid (System Biosciences). Following drug selection with puromycin and G418, several clonal lines were tested for their capacity to uniformly differentiate into neurons upon addition of 1 μg/ml doxycycline to the medium. A single *NGN2*-ESC line was used for all subsequent experiments. *NGN2*-ESCs were electroporated with the transposase plasmid along with one of five *piggyBac* vectors driven by a CMVe/synapsin promoter:tau[WT]-BioID2, tau[P301L]-BioID2, α-synuclein[WT]-BioID2, α-synuclein[A53T]-BioID2, or BioID2 only. The cells were expanded in zeocin-containing media for 3 weeks before the polyclonal BioID2 *NGN2*-ESC lines were ready for differentiation.

### Neuronal differentiation

Differentiation of *NGN2*-ESCs was performed according to Wang *et al.* (31), with some modifications. Briefly, to produce mature and terminally differentiated human neurons from *NGN2*-expressing hESCs, the cells were cultured in doxycycline to initiate differentiation and proliferation of neuronal precursors. This was followed by culture in terminal differentiation medium to generate mature and nondividing BioID2-expressing neurons for identification of interacting proteins by proximity biotinylation. The initial 5 days in culture expanded the numbers of immature neurons available for terminal differentiation and immediate use or for cryopreservation. Terminal differentiation in culture for 2 weeks or more produced mature nondividing neurons.

ESCs were dissociated with Accumax (Sigma) and plated (2 × 10^7^ cells/10 cm dish) on Matrigel in predifferentiation medium consisting of Dulbecco’s modified Eagle's medium/F12, doxycycline (1 μg/ml), nonessential amino acids (1×), laminin (0.2 μg/ml), brain-derived neurotrophic factor (10 ng/ml), neurotrophin-3 (10 ng/ml), and Y-27632 (10 μg/ml). The media were changed daily, and Y-27632 was removed after 48 h. On the fifth day of differentiation, the immature neurons were gently dissociated in Accumax and plated on Matrigel-coated 10 cm dishes (2 × 10^7^ neurons/dish) in 20 ml differentiation medium containing 1:1 Dulbecco’s modified Eagle's medium/F12:Neurobasal-A, nonessential amino acids, Glutamax (1×), doxycycline (1 μg/ml), laminin (1 μg/ml), B-27 supplement (0.5×), N-2 supplement (0.5×), brain-derived neurotrophic factor (10 ng/ml), and neurotrophin-3 (10 ng/ml). One-quarter of the medium was changed every week.

### Purification of biotinylated interactors

Two weeks after the final neuron plating, recombinant tau repeat-domain fibrils (K18[P301L]; 50 μg/dish), recombinant α-synuclein PFFs (50 μg/dish), or PBS alone was added to the medium. Biotin (50 μM; Sigma–Aldrich) was added at the same time as the fibrils or 3 weeks later. Twenty-four hours following the addition of biotin, the neurons were washed three times with PBS and lysed with 1 ml 8 M urea in 50 mM Tris, pH 7.4, or scraped and pelleted in PBS for detergent extraction. The lysates were frozen at −80 °C. Upon thawing, 1% Triton X-100 was added to the urea lysates followed by two rounds of sonication (60 s each) using a probe tip sonicator. Samples were centrifuged at 10,000*g* for 10 min, and the pellet was discarded. Streptavidin magnetic beads (200 μl; Pierce) were pre-equilibrated in 2 M urea + 0.25% Triton X-100 in 50 mM Tris, pH 7.4. The beads were mixed with 15 mg of lysate diluted with 2 M urea (4 ml total) and rotated overnight at 4 °C. The next day the beads were collected using a magnetic bead stand and extensively washed using the following series of buffers (1 ml each): once with 2 M urea in 50 mM Tris, pH 7.4; twice with 2% SDS in water; four times with PBS with Tween-20 + 850 mM sodium chloride + 1% sodium deoxycholate + 1% Triton X-100; three times with 50 mM Tris, pH 7.4 + 1 M lithium chloride + 1% sodium deoxycholate + 1% NP-40; once with water; and twice with 100 mM triethylammonium bicarbonate (TEAB), pH 8.0. Following the final wash, beads were resuspended in 50 μl TEAB.

### Sample preparation

Seven microliters of 0.5 M Tris(2-carboxyethyl)phosphine hydrochloride was added to the biotinylated interactors/streptavidin beads in 50 μl TEAB and incubated at 30 °C for 45 min in a thermomixer set at 1000 rpm. Five microliters of 0.5 M iodoacetamide was added to the samples and incubated for 30 min in the thermomixer at room temperature. Two micrograms of MS-grade trypsin/Lys-C mix (Promega) in 40 μl TEAB was added to each sample and incubated overnight in the thermomixer at 37 °C. The beads were removed by centrifugation, and the peptide-containing supernatant was quantified using a fluorescent peptide assay (Pierce). Peptides (25 μg) from each sample were labeled with TMT10plex reagents (Thermo Fisher Scientific) according to the manufacturer's instructions. The samples were combined and then purified using C18 stage tips, dried down, and resuspended in 15 μl for LC–MS.

### LC–MS and data analysis

MS experiments were performed using an Orbitrap Fusion Lumos instrument (Thermo Fisher Scientific) coupled with an UltiMate 3000 nano LC. Mobile phases A and B were water and acetonitrile, respectively, with 0.1% formic acid. One microliter of each TMT10plex sample (tau-BioID2 or α-synuclein-BioID2) was loaded directly onto an EASY-Spray PepMap RSLC C18 column (part number: ES803; Thermo Fisher Scientific) at a flow rate of 300 nl/min. All samples were separated using a linear gradient of 2 to 40% B over 120 min. MS data were acquired using a data-dependent acquisition-synchronous precursor selection MS (4) method for TMT quantification (71).

Survey scans of peptide precursors were performed from 375 to 1500 *m/z* at 120,000 full width at half maximum resolution with a 4 × 10^5^ ion count target and a maximum injection time of 50 ms. The instrument was set to run in top-speed mode with 3 s cycles for the survey and the MS/MS scans. After a survey scan, tandem MS was then performed on the most abundant precursors exhibiting a charge state from 2 to 7 of greater than 5 × 10^4^ intensity by isolating them in the quadrupole at 0.7 Da. Collision-induced dissociation fragmentation was applied with 35% collision energy, and resulting fragments were detected in the ion trap. The maximum injection time was limited to 50 ms, and dynamic exclusion was set to 60 s with a 10 ppm mass tolerance around the precursor. TMT reporter ions were quantified in MS3 using synchronous precursor selection of ten notches from the MS2 spectrum with higher energy collisional dissociation fragmentation collision energy at 65%, Orbitrap resolution at 120,000, and a maximum injection time of 246 ms.

Peptides were identified using the SEQUEST HT search algorithm (Thermo Fisher Scientific) within Proteome Discoverer (version 2.3; Thermo Fisher Scientific). MS2 spectra were matched against a *Homo sapiens* proteome database (SwissProt TaxID: 9606, version 2017-10-25) using an FDR of <1%. The following search parameters were used: MS1 precursor tolerance = ±10 ppm, MS2 fragment mass tolerance = ±0.6 Da, MS3 reporter ion tolerance = ±20 ppm, and maximum missed cleavages = 2. Carbamidomethylation at cysteine was treated as a static modification (+57.0215 Da). Dynamic modifications included TMT labels at the peptide N terminus and lysine (+229.1629 Da), oxidation at methionine (+15.9949 Da), biotin at lysine (+226.0776 Da), and acetylation at the protein N terminus (+42.0367 Da). The data from three injections were combined, and the reporter ion abundances were averaged for each sample. We considered the identified proteins to be tau-interacting or α-synuclein–interacting proteins if they met the following conditions: the peptides were detected in at least two of three injections, the number of PSMs was ≥2, and the mean normalized abundance in fibril-treated or fibril-untreated samples was ≥1.5-fold higher than in both BioID2-only control samples (24 h and 3 weeks). The abundance plots and heat maps were generated using Proteome Discoverer 2.3.

### Detergent extraction

Neurons harvested in PBS were lysed in ice-cold 1% Triton X-100 + 150 mM sodium chloride in 50 mM Tris and sonicated twice for 60 s using a probe tip sonicator. The samples were centrifuged at 100,000*g* for 1 h. The protein concentration in the supernatant was quantified by bicinchoninic acid assay, and the pellet was washed in lysis buffer before resuspending in 1× NuPAGE LDS sample buffer + NuPAGE reducing agent (Thermo Fisher Scientific) and boiled for 10 min. A volume equivalent to 10 μg of the soluble fraction was loaded for immunoblot analysis. Anti-rabbit–HRP secondary was used at 1:10,000 dilution.

### Western blotting

Urea-solubilized neuron lysates were mixed 1:1 with 2× NuPAGE LDS sample buffer and boiled for 10 min. About 30 μg of each sample were run on 12% Bis–Tris gels and transferred to polyvinylidene fluoride membranes. The blots were probed with primary antibodies including antitotal tau (1 μg/ml; tau12) and antitotal α-synuclein (1:1000 dilution; Syn211). Detergent-extracted samples were probed using polyclonal antibodies generated in-house from a rabbit immunized with a synthetic peptide corresponding to the R2 region of tau (4R specific). Anti-mouse-HRP or anti-rabbit-HRP secondary antibodies were used at 1:10,000 dilution (Thermo Fisher Scientific). Mouse anti-actin-HRP (1:2000 dilution; Novus) was used as a loading control. For detection of biotinylated proteins, the blots were blocked with 2.5% bovine serum albumin in Tris-buffered saline plus Tween-20, probed with streptavidin-HRP (1:25,000 dilution; Abcam; catalog no.: ab7403) for 1 h, blocked with 10% fetal bovine serum, and then washed with Tris-buffered saline plus Tween-20 prior to developing.

### Immunofluorescence

Neurons were plated (30,000 neurons/well) for terminal differentiation in Matrigel-coated 96-well plates (CELLSTAR Greiner Bio-One). After 6 weeks of terminal differentiation, the neurons were fixed with 4% paraformaldehyde/4% sucrose in PBS, permeabilized with 0.3% Triton X-100, and blocked with 5% goat serum. The neurons were incubated with the following primary antibodies overnight: anti-MAP2 (1:5000 dilution; Sigma; catalog no.: PA1-10005); anti-synaptophysin (1:500 dilution; Abcam; catalog no.: YE269); anti-tubulin, beta III isoform (1:1000 dilution; Millipore; catalog no.: MAB1637); anti-total tau (1 μg/ml; tau5); anti-phospho-tau (1:200 dilution; Thermo Fisher Scientific; catalog no.: AT8); anti-total α-synuclein (1:200 dilution; Thermo Fisher Scientific; catalog no.: Syn211); anti-phospho-α-synuclein pS129 (1:200 dilution; Abcam; catalog no.: EP1536Y); or anti–vesicular glutamate transporter 1 (1:1000 dilution; Thermo Fisher Scientific; catalog no.: 48-2400). Primary antibody incubation was then followed by incubation with secondary antibodies for 2 h: goat anti-rabbit, anti-mouse, or anti-chicken conjugated to Alexa Fluor 488, 568, or 647 (1:250 dilution; Thermo Fisher Scientific). Images were acquired using a Leica SP8 confocal microscope.

### Expression and purification of recombinant tau and α-synuclein

*Escherichia coli* BL21-CodonPlus (DE3)-RP (Agilent) was transformed with a pET28a plasmid encoding WT or P301L tau (full-length 0N4R). Terrific broth cultures (1 l) supplemented with 50 mg/l kanamycin or 50 mg/l chloramphenicol were inoculated with 20 ml of starter cultures and grown for 8 h. The cultures were induced with 1 mM IPTG and grown for another 16 h. Cells were harvested and resuspended in 50 ml/l 20 mM MES, pH 6.8, 1 mM EGTA, 1 mM magnesium chloride, 5 mM DTT, and 1 cOmplete protease inhibitor cocktail (Roche) followed by microfluidizer lysis. The lysates were boiled for 20 min and centrifuged at 48,400*g*. The cleared lysates were applied to a cation exchange column (SP Sepharose Fast Flow; GE Healthcare), and fractions were eluted with a sodium chloride gradient. Fractions containing 0N4R tau were applied to a reversed-phase HPLC column and eluted with an acetonitrile gradient (1%/min) + 0.1% TFA gradient; the peak fractions were then lyophilized. The lyophilizates were dissolved in PBS + 1 mM DTT and purified by size-exclusion chromatography (HiLoad 26/600 Superdex 200 pg; GE Healthcare). Peak fractions were analyzed by SDS-PAGE, and fractions containing <95% 0N4R tau were pooled, snap-frozen, and stored at −80 °C. Recombinant full-length α-synuclein and tau repeat domain (K18) were expressed and purified as previously described (72, 73).

### *In vitro* fibrillization and RT-QuIC

α-Synuclein PFFs were generated according to Volpicelli-Daley *et al.* (17). K18[P301L] (residues 244–372) tau fibrils were generated with heparin as previously described (74, 75, 76). Following fibrillization, fibers were collected by centrifugation to remove nonfibrillar species. The protein concentration in the fibrillar sample was determined spectrophotometrically. Tau RT-QuIC was performed as previously described (77) with some modifications. Recombinant 0N4R tau (WT or P301L) was fibrillized in 1.5 ml Lo-bind tubes and included Dulbecco’s PBS, 10 μM tau, 10 mM DTT, and 1× HALT protease inhibitor cocktail (without EDTA; Thermo Fisher Scientific). Heparin (25 μg/ml; molecular weight = 8000–25,000; Santa Cruz) and recombinant His-tagged 14-3-3 proteins (ƞ, θ, or ζ; 2 μM; Enzo) were added to some reactions. The molar ratio of tau:heparin was approximately 10:1. The mixtures were shaken at 37 °C and 900 rpm (30 s on/off cycles) for 7 days (FLUOstar Omega; BMG Labtech). ThT fluorescence measurements (450 nm/480 nm) were taken every 15 min.

### STRING interaction analysis

The list of gene names corresponding to significantly enriched protein interactors shared by both tau and α-synuclein 3 weeks postseeding (≥1.5-fold more abundant than both BioID2-only controls) was compared with a reference interactome to construct a network of enriched interactions using STRING interaction analysis (39). The network was generated and visualized using the StringApp in Cytoscape, version 3.8 (https://cytoscape.org/) with default settings (78, 79).

### GO analysis

Data represent the mean ± SD unless otherwise noted. Statistical analyses were performed with PANTHER, version 15.0, and Prism 6 (GraphPad Software, Inc). The molecular functions of identified WT tau and α-synuclein interactors at 3 weeks (+/− K18 or +/− PFF combined list) were determined by a statistical over-representation test (www.pantherdb.org) (38). The top ten nonredundant GO terms (molecular function complete) with an FDR ≤0.05 were plotted according to their fold-enrichment relative to a reference human gene set.

## Data availability

The MS-based proteomics data have been deposited to the ProteomeXchange Consortium *via* the MassIVE partner repository and are available with the identifier PXD035102.

## Supporting information

This article contains supporting information.

## Conflict of interest

The authors declare that they have no conflicts of interest with the contents of this article.
